# Simvastatin impairs hippocampal synaptic plasticity and cognitive function in mice

**DOI:** 10.1186/s13041-021-00758-x

**Published:** 2021-02-24

**Authors:** Yujun Guo, Guichang Zou, Keke Qi, Jin Jin, Lei Yao, Yang Pan, Wei Xiong

**Affiliations:** 1 Department of Neurosurgery, Institute On Aging and Brain Disorders, The First Affiliated Hospital of USTC, Division of Life Sciences and Medicine, Hefei National Laboratory for Physical Sciences At the Microscale, University of Science and Technology of China, Hefei, 230026 China; 2 National Synchrotron Radiation Laboratory, University of Science and Technology of China, Hefei, 230029 China; 3 Advanced Innovation Center for Human Brain Protection, Capital Medical University, Beijing, 100070 China; 4 Center for Excellence in Brain Science and Intelligence Technology, Chinese Academy of Sciences, Shanghai, 200031 China

**Keywords:** Simvastatin, Cholesterol, Hippocampus, Cognition, Mass spectrometry imaging

## Abstract

Lipophilic statins which are blood brain barrier (BBB) permeable are speculated to affect the cholesterol synthesis and neural functions in the central nervous system. However, whether these statins can affect cholesterol levels and synaptic plasticity in hippocampus and the in vivo consequence remain unclear. Here, we report that long-term subcutaneous treatments of simvastatin significantly impair mouse hippocampal synaptic plasticity, reflected by the attenuated long-term potentiation of field excitatory postsynaptic potentials. The simvastatin administration causes a deficiency in recognition and spatial memory but fails to affect motor ability and anxiety behaviors in the mice. Mass spectrometry imaging indicates a significant decrease in cholesterol intensity in hippocampus of the mice receiving chronic simvastatin treatments. Such effects of simvastatin are transient because drug discontinuation can restore the hippocampal cholesterol level and synaptic plasticity and the memory function. These findings may provide further clues to elucidate the mechanisms of neurological side effects, especially the brain cognitive function impairment, caused by long-term usage of BBB-permeable statins.

## Introduction

Statins are the most effective low density lipoprotein-cholesterol lowering medications by targeting 3-Hydroxy-3-methylglutaryl coenzyme A (HMG-CoA) reductase in blood and liver [1, 2]. Statins have widely been recognized as the first-line medications for the therapy of strokes and cardiovascular diseases for years [3, 4]. Various types of statins including atorvastatin, lovastatin, rosuvastatin and simvastatin have been approved by the U.S. Food and Drug Administration (FDA) [5]. According to their capacity to cross the blood–brain barrier (BBB), statins are classified as lipophilic statins including atorvastatin, simvastatin and lovastatin which are BBB-permeable, and hydrophilic statins including rosuvastatin and pravastatin which are BBB-impermeable [6]. The lipophilic simvastatin has been reported to significantly reduce brain cholesterol level in mice, when compared with hydrophilic pravastatin [7]. Clinical studies have also shown that atorvastatin and simvastatin usage could cause reversible cognitive function impairment [8, 9]. However, the underlying mechanisms upon how statins affect the brain cognitive function remain unsolved.

Cholesterol is ubiquitous in the central nervous system (CNS). Accurate maintenance of brain cholesterol level is essential for normal brain function including signaling and synaptic plasticity [10, 11]. Brain cholesterol metabolic deficiency has been linked to varieties of neurological disorders, such as Alzheimer’s disease, Parkinson’s disease and Huntington disease [12–14]. Human studies have demonstrated that low levels of total cholesterol are associated with poor performance on cognitive function [15]. Animal studies also indicated that animals with cholesterol synthesis deficiency suffer severe declines in learning and memory abilities [16, 17]. Dietary cholesterol can improve performance of rodents in Morris Water Maze (MWM) tests. Such improvement is suggested to be associated with the changes in synaptic plasticity of hippocampus [18, 19].

Hippocampal synaptic structure and function are always linked to brain cognition [20, 21]. Hippocampal cholesterol loss may impair brain synaptic functions including electrical or chemical signal transmission and therefore may lead to the poor cognition [22–25]. Although BBB-permeable statins have been suggested to affect brain cognition, it remains unclear whether they affect cholesterol levels in hippocampus and the hippocampal synaptic plasticity. To answer these questions, here we combined our recently developed desorption electrospray ionization mass spectrometry with photoionization assistance (paDESI-MS) imaging technology [26] with field potential recordings and behavioral tests. Chronic simvastatin treatments indeed significantly reduced long-term potentiation (LTP) in hippocampal slices of mice and impaired their recognition memory. The MS imaging revealed a remarkable down-regulation of cholesterol in hippocampus in simvastatin-treated mice. Furthermore, drug withdrawal significantly restored the hippocampal synaptic plasticity and the memory function of mice, with simultaneous recovery of cholesterol level in the hippocampus. These findings provide a basis for studying the neurological and cognitive side effects of BBB-permeable lipophilic statins.

## Methods

### Animals

All procedures have been approved by the Institutional Animal Use and Care Committee of School of Life Sciences, University of Science & Technology of China. Adult C57BL/6J male mice at 5 weeks of age were obtained from Vital River Laboratory Animal Technology Co., Ltd. (Beijing, China). After acclimating for a week, mice received administration of simvastatin (S.C., 30 mg/kg) or vehicle for 26 consecutive days. All behavioral tests were performed from Day 21 to Day 26. All mice were housed at 18–23 ℃ with 40–60% humidity under a 12-h dark/light cycle (lights off at 7 p.m) and free access to food and water.

### Morris water maze (MWM)

After receiving 20 consecutive days (Day 1-Day 20) of vehicle/simvastatin treatments, the mice were arranged for the MWM tests (Day 21). Mice of each group were trained in a large tank (120 cm in diameter and 40 cm in depth) which was divided into four quadrants. A hidden 10-cm-diameter platform (1 cm below the surface of water) was placed in the center of a quadrant. The pool was surrounded by a black curtain with four visual cues on the wall of pool. Water was kept at 20° C and opacified with titanium dioxide. The trials were conducted 4 times daily at the same time point for 5 successive days followed by a probe test on Day 6. Mice were placed into four quadrants in order (20 min interval) and swam freely for a maximum of 60 s. If a mouse did not find the platform within a 60-s period, it was gently guided to the platform and allowed to stay on the platform for 15 s. The latency, distance and speed of mice to find platform were recorded. For probe test, the platform was removed from the pool and the mouse was put into the quadrant opposite to where the platform located and allowed to swim for 30 s. The time of the mice spent in each quadrant was recorded.

### Novel object recognition (NOR)

After receiving 20 consecutive days of vehicle/simvastatin treatments, another group of mice were arranged for the NOR tests (Day 21–22). The open-field apparatus consisted of an acrylic chamber (40 cm × 40 cm × 30 cm). Two different objects were prepared in duplicate: towers of rectangular Lego bricks (built from blue, green and yellow bricks) and circular Lego bricks (built from yellow and red bricks). The objects were placed 10 cm away from the walls and attached to the floor. Mice were tested in the dark (active phase between 7:00 p.m. and 7:00 a.m.). During the familiarization session, mice were allowed to freely explore two identical objects (rectangular Lego) placed into the arena at fixed locations for 3 min. The ANY-maze video-tracking system (Stoelting, Wood Dale, USA), which is based on nose-point detection, was used to record the time mice spent exploring objects. Active exploration was defined as mice sniffing or touching the object when the gap between the nose and the object was less than 2 cm. Climbing over the object or gnawing the object was not considered as exploratory activity. At the end of the test, each mouse was returned to its home cage, and the chamber and objects were cleaned using 75% ethanol, then air-dried for 3 min. The mice with no significant preference for the two identical objects were selected for further tests. In the NOR tests, 6 of 34 mice were excluded based on their abnormal preference to specific legos. After an intersession interval (ISI) of 24 h, one of the familiar objects was replaced by a novel object (circular Lego). The location of the novel object (left or right) was randomized among the mice and the groups tested. Object preference was calculated by using the following formula: preference % = (time to explore the individual object/total exploration time for both objects) × 100%. Data were excluded if the total of exploration time was less than 10 s. After the novel object recognition test, mice were allowed to recover for 2 days before further behavioral tests.

### Open field test (OFT)

The open field test was performed 2 days after the NOR test (Day 24). An open field test system (XR-XZ301, Xinruan, Shanghai, China) was used. Mice were individually transferred from their home cages to an open field chamber (width, 45 cm; length, 45 cm; height, 45 cm) for locomotion tests for 15 min. Locomotor activity was recorded by a camera and the distance each mouse travelled was analyzed by the ANY-MAZE software (Global Biotech Inc.).

### Rotarod test (RT)

The rotarod test was performed on the next day after the OFT (Day 25). A rotarod training system (XR1514, Xinruan, Shanghai, China) was used. Before the first training session, mice were habituated to stay on a stationary rod for 2 min. A total of six trials for the rotarod test were carried out using an accelerating protocol from 4 to 60 rpm in 300 s with 20-min inter-trial intervals. After falling, the mice were immediately placed back to their home cages and the time to fall was automatically recorded by the rotarod software. Once the trial reached to 300 s, the mice were manually removed from the rod immediately. The apparatus and testing area were cleaned with 75% ethanol (w/v) after each trial.

### Elevated plus maze (EPM)

The elevated plus maze was performed on the next day after RT (Day 26). The EPM apparatus consisted of a cross-shaped maze (with 25 cm × 5 cm arms) elevated by a 60-cm support. Two opposite arms were surrounded by a 20-cm wall, while the other two were open (only with a 1-cm contention step). Mice were individually placed in the central area of the apparatus, facing one of the closed arms, and their mobility within the maze was assessed over 5 min. The exploration profile within the different areas of the maze (open arms, closed arms and center) was analyzed. The anxiety behavior was assessed by examination of the open arm exploration. Animals that fell from the apparatus had to be censored from the analyses. Arm preference was automatically analyzed by the ANYmaze video tracking software.

### Hippocampal slice preparations and electrophysiological recordings

The mice were sacrificed on the next day after all behavioral tests were finished (Day 27). Coronal hippocampal slices (350-μm thick) from adult male mice were prepared with Leica Vibratome in ice-cold cutting solution containing (in mM) 30 NaCl, 26 NaHCO_3_, 10 Glucose, 194 sucrose, 4.5 KCl, 1.2 NaH_2_PO_4_, 1 MgCl_2_ and continuously bubbled with carbogen (95% O_2_ + 5% CO_2_). The slices were then recovered at room temperature for 1 h. Slices were transferred into the recording chamber continuously perfused at 12 ml/min with artificial cerebrospinal fluid (ACSF) at 37 ℃. The constituent of ACSF are the followings: (in mM): 124 NaCl, 4.5 KCl, 1 MgCl_2_, 2 CaCl_2_, 1.2 NaH_2_PO_4_, and 26 NaHCO_3_, continuously bubbled in carbogen. Long-term potentiation (LTP) was triggered by high frequency stimulations (HFS, 100 Hz, 1 s) in the hippocampal CA3 area. Field excitatory postsynaptic potentials (fEPSPs) were recorded using a glass electrode (filled with NaCl, 3–6 MΩ) placed into the stratum radiatum of the CA1 area. Signals were amplified (gain 100) and filtered (3 kHz), then digitized (10–100 kHz; National Instruments). After a 20-min baseline recording, recordings were continued for at least 50 min following LTP induction. The LTP was quantified by the fEPSP slope normalized to the baseline. Paired-pulse ratio (PPR) was obtained by delivering two stimulation pulses with interstimulus intervals of 50 ms. PPR values were quantified by calculating the ratio between the mean amplitude of the second and the first fEPSP. Synaptic responses were evoked at 0.1 Hz using a bipolar tungsten electrode. Data were collected and analyzed on or off-line by using pClamp 10.4 software (Molecular Devices, Sunnyvale, CA) software.

### paDESI-MS imaging

The mice used for paDESI-MS imaging also received vehicle/simvastatin treatments and behavioral tests except those in Fig. 3c and were then sacrificed on the next day after behavioral tests (Day 27). The brain was immediately removed from the skull and flash frozen in liquid nitrogen for 15 s. The frozen mouse brain was transferred to the cryostat chamber of a Vibratome (VT 1200S, Leica, Germany) at − 20 °C. Brains from vehicle group and simvastatin group were separately cut into 16-μm-thick coronal sections. In each group, three adjacent hippocampal slices were collected for parallel experiments. One slice from control group and one slice from simvastatin group were placed on the same microscope slide to avoid the matrix effects caused by different slides. The slide was then scanned by paDESI-MS. The cholesterol intensity was normalized to ^13^C3-cholesterol (0.1 mg/mL) which has been added into the spray. The major fragment of cholesterol is at m/z = 369.3532 [M − H_2_O + H] + and the major fragment of ^13^C3-cholesterol is at m/z = 372.3628. Thus, we can examine the cholesterol intensity semi-quantitatively by normalizing the brain cholesterol to the signal intensity of [M − H_2_O + H] + ions of ^13^C3-cholesterol. The changes in cholesterol were calculated as changes in cholesterol = ((C − Cmean)/Cmean)*100. C represents the normalized cholesterol level (normalized to the signal intensity of ^13^C3-cholesterol of the hippocampus and Cmean represents the mean of the normalized cholesterol levels in the hippocampus of vehicle-treated mice.

paDESI-MS imaging system consisted of a DESI sprayer, a 2D scanning stage, and a postphotoionization interface. A solvent was infused at a flow rate of 3 μL/min through a DESI sprayer (50 μm i.d. and 150 μm o.d. inner fused silica capillary and a 250 μm i.d. and 350 μm o.d. outer fused silica capillary) and directed onto the surface of a tissue slice with a 53° angle of incidence with the assistance of the nebulizing N_2_ gas (120 psi). The flow of the solvent was driven by a syringe pump, and the metal needle tip was connected to a high-voltage power supply (3500 V for the positive ion mode and − 4000 V for the negative ion mode). The desorbed compounds were sucked in the heated transfer tube (i.d. 0.5 mm, o.d. 1/16in.) with a 10° angle of collection, and the un-ionized neutral molecules were ionized in an ionization tube (i.d. 4 mm, o.d. 10 mm) by a coaxially oriented krypton DC discharge vacuum ultraviolet (VUV) lamp, which was positioned to shine toward the exit of the transfer tube. Then the ionized species was transferred into a capillary of mass spectrometer. In order to improve the transfer efficiency, an air-flow assisted transport arrangement was added in this interface, and a pneumatic diaphragm pump (60 L/min, model GM-1.0A, Jinteng Experimental Equipment Co., Ltd., Tianjin, China) was connected to the side port of the ionization tube. In experiments, the transfer tube and ionization tube were kept at 300 °C. Note that the krypton lamp was turned off in the DESI mode and turned on in the DESI/PI mode. All imaging data were collected on an Agilent 6224 Accurate-Mass TOF mass spectrometer (Agilent, USA). The flow rate and temperature of drying gas of the mass spectrometer were set at 5 L/min and 325 °C, respectively. A programmable motorized X–Y scanning stage (GCD-203050 M, Daheng, Beijing, China) was used for tissue imaging, and the scanning process was allowed to be synchronized with the Agilent mass spectrometer data acquisition by the customized stage control software. The sample surface was line scanned in the X direction with a stepper motor at a velocity of 370 μm/s while acquiring mass spectra every 0.5 s. The distance between adjacent scan lines in the Y direction was 200 μm. The acquired multiple scan lines were combined in one data file for ion distribution images by using the freely available standalone version of the MSiReader software.

For simvastatin discontinuation experiments, the control group and simvastatin group received 26-day vehicle or simvastatin treatments and were then sacrificed on Day 27. The brain was then removed and frozen at − 80 °C for further MS imaging. The discontinuation group suffered 4-week simvastatin discontinuation after 26-day simvastatin treatments. After the discontinuation session, the mice were sacrificed and the brain was removed and frozen at − 80 °C. Brains from vehicle group, simvastatin group and simvastatin discontinuation group were separately cut into 16-μm-thick coronal sections. In each group, three adjacent hippocampal slices were collected for parallel experiments. One slice from control group, one slice from simvastatin group and one slice from simvastatin discontinuation group were placed on the same microscope slide to avoid the matrix effects caused by different slides. The slide was then scanned by paDESI-MS. The identifications for most of these peaks were facilitated by accurate m/z values, comparison of isotope distribution patterns, and tandem mass spectrometry.

### Statistics

All experiments and data analysis were conducted in a blinded way. All statistical analyses for in vitro recording and behavioral experiments were performed using Prism7 software (GraphPad). Data were statistically compared by unpaired t tests, as indicated in the specific figure legends. Average values are expressed as the mean ± SEM. P < 0.05 was considered significant.

## Results

### Hippocampal LTP is inhibited in simvastatin-treated mice

**Fig. 1:**
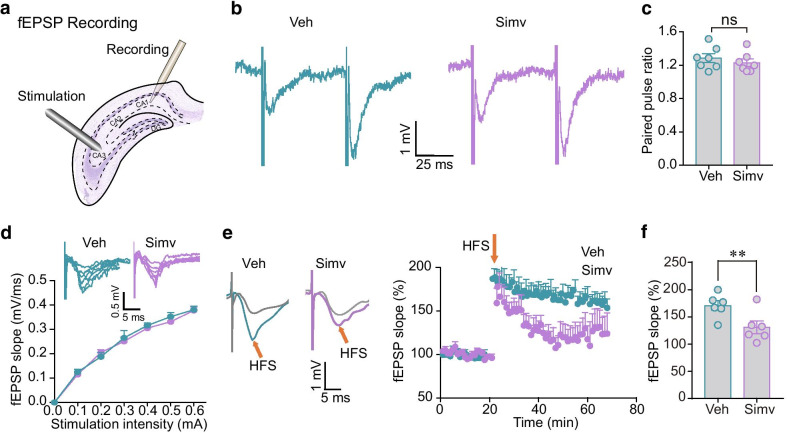
Effects of simvastatin on hippocampal LTP. **a** Schematic of hippocampal fEPSP recording. **b** Representative traces of fEPSPs recorded in CA1 from vehicle- and simvastatin-treated mice following paired-pulse stimulation protocol (interstimulus interval 50 ms). **c** Average values of paired-pulse ratio in CA1 from vehicle- and simvastatin-treated mice. n = 7 from 3 mice. **d** Input–output curves constructed from the stimulation intensity vs fEPSP slope in the hippocampus from brain slices of vehicle- and simvastatin-treated mice. Inlet: sample traces for the input output curves. n = 6 from 3 mice. **e** Left: representative traces of fEPSPs evoked by electrical stimulation of the Schaffer-commissural projection before and after HFS stimulation in mice treated with chronic vehicle or simvastatin (S.C., 30 mg/kg/day, 26 days). Right: time course of fEPSP slope normalized to baseline. The fEPSP slope is plotted as a percentage change against the baseline (0–20 min) before HFS. HFS: high frequency stimulation. HFS is indicated by arrows. **f** Normalized fEPSP slope values (as a percentage of the baseline) in mice treated with chronic vehicle or simvastatin. n = 6 slices from 3 mice per group. Data are represented as mean ± SEM. **P < 0.01 based on an unpaired t test; ns, not significant (P > 0.05)

First, we examined the LTP, a main form of synaptic plasticity that underlies synaptic information storage within the CNS [27], in the hippocampal slices of mice receiving chronic subcutaneous (S.C.) simvastatin administration (30 mg/kg/day, 26 days). Field excitatory postsynaptic potentials (fEPSPs) were recorded in CA1 area in response to the electrical stimulation of Schaffer commissural pathway (Fig. 1a). After setting of stimulating and recording electrodes into hippocampal CA3 and CA1, an input–output curve was constructed by stimulating at intensities ranging from 0 to 0.6 mA. Before LTP recording, we assessed the effects of simvastatin on presynaptic function of CA1 using a paired-pulse ratio (PPR) test. The results showed that simvastatin-treated mice showed a similar PPR compared with vehicle-treated mice, suggesting that the presynaptic release probability is unchanged (Fig. 1b, c). We then examined whether the basal synaptic field responses in the hippocampus were altered by simvastatin, by comparing input–output curves constructed from the stimulation intensity vs fEPSP slope. No significant differences between vehicle- and simvastatin-treated mice in the overall input–output curves were observed (Fig. 1d). These results suggest that long-term treatment of simvastatin does not affect the basal synaptic transmission. We next investigated whether simvastatin would affect synaptic plasticity induced by HFS. High frequency stimulation (HFS, 100 Hz, 1 s) was used to achieve LTP, before which a 20-min baseline recording was performed. The HFS-induced potentiation of fEPSP was significantly reduced in the simvastatin-treated mice when compared with the vehicle-treated mice (Fig. 1e, f). These results indicate that chronic simvastatin usage may impair the hippocampal synaptic plasticity.

### Chronic simvastatin treatments impair recognition and spatial memory

**Fig. 2:**
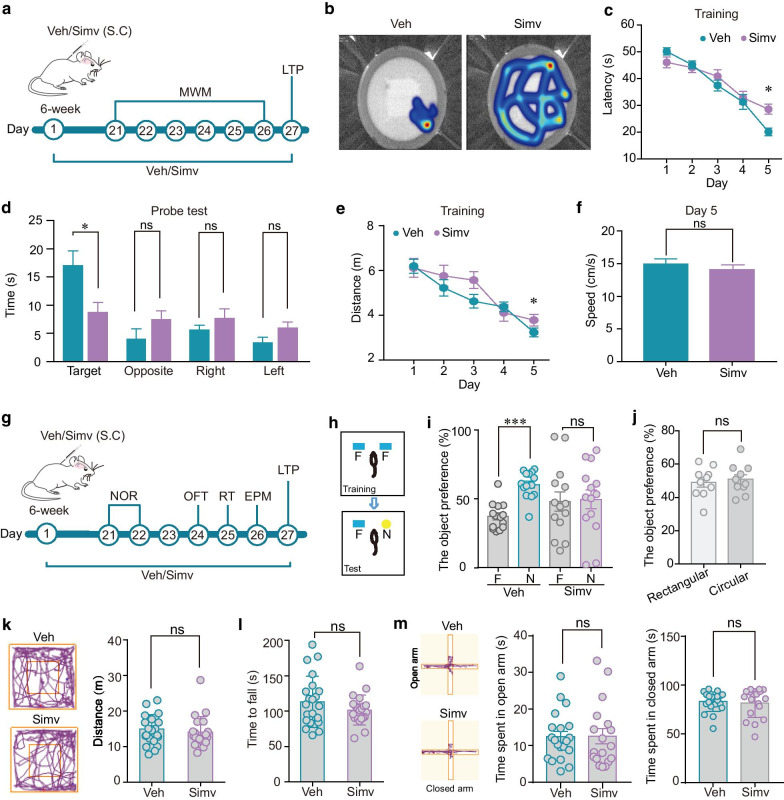
Effects of simvastatin on neurological behavioral performance. **a** Schematic diagram showing simvastatin treatments, MWM behavioral tests and LTP recordings. MWM: Morris water maze. Veh/Simv: Vehicle/Simvastatin. S.C: Subcutaneous. The simvastatin treatment on the 6-week old mice started on Day 1 and the MWM test was performed after 21-day simvastatin treatments (S.C., 30 mg/kg/day). **b** Representative heat map traces of mice in the MWM test. **c** Time spent in finding the platform in mice receiving chronic vehicle or simvastatin treatments. n = 8 per group. **d** Average values of time spent in the target quadrant and the other three quadrants (opposite, right and the left) during the probe test. n = 8 per group. **e**, **f** Average values of swimming distance in finding the platform (**e**), and swimming speed (**f**), in mice receiving chronic vehicle or simvastatin treatments. n = 8 per group. **g** Schematic diagram showing simvastatin treatments, various behavioral tests and LTP recordings. NOR: Novel object recognition; EPM: Elevated plus maze; OFT: Open field test; RT: Rotarod test. **h** Schematic diagram of the NOR test. **i** Average values of time the vehicle- and simvastatin-treated mice spent in exploring the familiar (F) and the novel objects (N). n = 14 per group. **j** Average values of time the mice without NOR training spent in exploring the rectangular Lego and circular Lego. n = 10 per group. **k** Left: representative traces of mice travelling in the open field test. Right: average values of the travelling distance of the vehicle- and simvastatin-treated mice in the open field. n = 21–22. **l** Average values of the latency to fall on the rotarod of the vehicle- and simvastatin-treated mice. n = 22 per group. **m** Left: representative traces of mice moving along the elevated plus maze. Right: average values of the time that the vehicle- and simvastatin-treated mice spent in the open arms and closed arms of the elevated plus maze. n = 18–20. Data are represented as mean ± SEM. *P < 0.05, ***P < 0.001 based on unpaired t tests; ns, not significant (P > 0.05)

We next conducted behavioral tests including novel object recognition (NOR) and Morris water maze (MWM) to examine the effects of simvastatin on the development of recognition and spatial memory, both greatly involving the hippocampal synaptic plasticity (Fig. 2a, g).

For the MWM test, mice were required to find a hidden platform to escape from swimming in a pool of water. The pool contained four quadrants and the mice were placed into four quadrants orderly (20-min interval) to swim freely for a maximum of 60 s. Four consecutive trials were conducted daily at the same time point for five successive days from Day 1 to Day 5. The simvastatin-treated mice showed an increased latency to find the platform compared with vehicle-treated mice on Day 5 (Fig. 2b, c). Additional probe trials demonstrated that simvastatin-treated mice spent less time in the target quadrant than the vehicle-treated mice (Fig. 2d). Similarly, simvastatin-treated mice also travelled a long distance compared with vehicle-treated mice on Day 5 (Fig. 2e). These results showed that long-term simvastatin treatments may cause deficiency in spatial memory. Such impairment seems to be independent of the swimming ability and sensitivity to water because the swimming speed was unchanged in simvastatin-treated mice (Fig. 2f).

For the NOR tests, the vehicle- and simvastatin-treated mice were adapted to the training room for 30 min. Then, the mice were allowed to freely explore two identical objects (rectangular lego) placed into the arena at fixed locations for 3 min. The mice with no significant preference for the two identical objects were selected for further tests. After an intersession interval (ISI) of 24 h, one of the original objects was replaced by a novel object (circular lego) and the object preference was calculated (Fig. 2h). The vehicle-treated mice spent more time exploring the novel object compared with the familiar object. Such preference to the novel object was significantly inhibited in the simvastatin-treated mice, indicating a deficiency in recognition memory (Fig. 2i). Such deficiency in memory is certainly not due to the preference of mice to the shape of lego itself (Fig. 2j).

We further examined the effects of simvastatin on other neurological behaviors. Simvastatin did not affect locomotor activity and motor coordination of mice, reflected by unchanged travel distance in the open field test and unaltered time to fall in the rotarod test (Fig. 2k, l). In the elevated plus maze test, time spent in the open and closed arms was not changed in the simvastatin treated mice compared with the vehicle-treated mice (Fig. 2m).

### Chronic simvastatin treatments reduce cholesterol levels in hippocampus

**Fig. 3:**
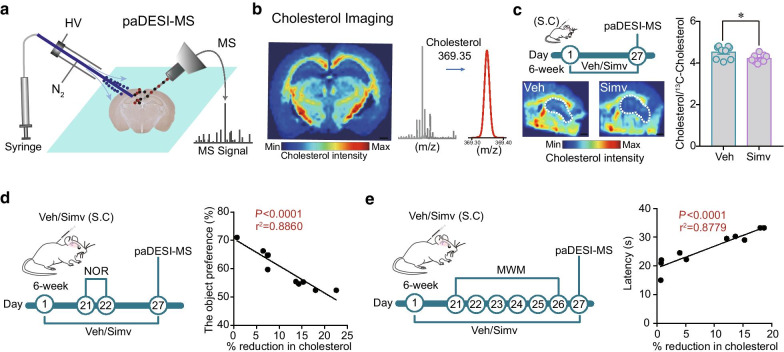
Effect of chronic simvastatin treatments on hippocampal cholesterol level. **a** Schematic diagram of paDESI-MS imaging setup. HV, high voltage; N_2_, nitrogen; MS: mass spectrometry. **b** Representative brain cholesterol image and MS spectra of cholesterol obtained from the brain slice. **c** Representative cholesterol images of brain slices from the vehicle- and simvastatin-treated mice. Normalized brain cholesterol intensity in the vehicle- and simvastatin-treated mice. n = 7–9. Scale bar: 1 mm. Simv: simvastatin, Veh: vehicle. Data are represented as mean ± SEM. *P < 0.05 based on an unpaired t test. **d** Schematic diagram showing simvastatin treatments, NOR behavioral tests and paDESI-MS imaging in the same mice. Correlation analysis of the simvastatin-induced cholesterol reduction (% reduction vs control group) in the hippocampus of mice and their novel object preference (%) (n = 10). **e** Schematic diagram showing simvastatin treatments, MWM behavioral tests and paDESI-MS imaging. Correlation analysis of the extent of simvastatin-induced cholesterol reduction in hippocampus and the latency in finding the platform (n = 10). The latency data at the last day of MWM training was shown. All the dots corresponding to individual mice were randomly selected from the simvastatin-treated group. n = 10 mice

To examine whether long-term usage of the BBB-permeable simvastatin affects the hippocampal cholesterol level, we used our recently developed paDESI-MS imaging technique [26] to quantify the intensity of cholesterol in the hippocampus of mouse brain sections (Fig. 3a, b). The paDESI-MS technique combines conventional DESI with a postphotoionization. The advantage of this technology is that it enhances the ionization and imaging of desorbed neutral molecules such as cholesterol in biological tissue sections. Considering that it will take a long time for paDESI-MS to scan a whole brain slice and such a long time may cause degradation of metabolites, in this study we only screened and analyzed a small brain area containing the hippocampus (Fig. 3c). Long-term simvastatin administration significantly reduced brain cholesterol concentration in the hippocampus of mice. There was a strong correlation between hippocampal cholesterol intensities with the recognition memory (Fig. 3d) and the spatial memory of mice (Fig. 3e). Taken together, these results suggest that the simvastatin-induced synaptic plasticity impairment and cognition deficiency are correlated with the down-regulation of cholesterol level in hippocampus.

### Simvastatin discontinuation restores hippocampal cholesterol levels, synaptic plasticity and memory

**Fig. 4:**
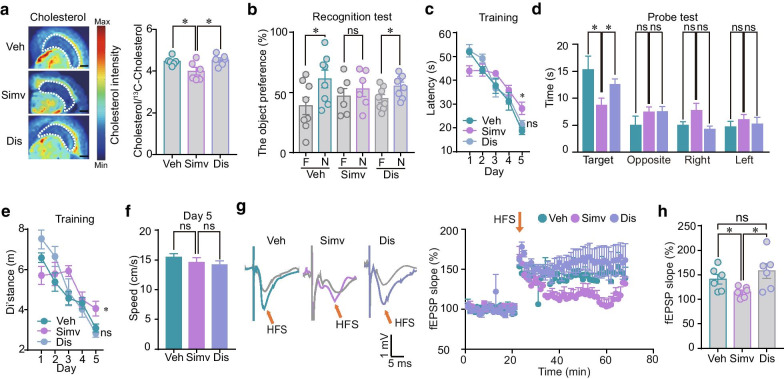
Effects of simvastatin withdrawal on hippocampal cholesterol levels, memory behaviors and hippocampal LTP. **a** Representative images and normalized intensity of hippocampal cholesterol in the vehicle (Veh)- and simvastatin (Simv)-treated mice and the mice with simvastatin discontinuation (Dis). n = 6 per group. Scale bar: 1 mm. **b** Average values of time the different groups of mice spent in exploring the familiar (F) and the novel objects (N) in the NOR tests. n = 6–8. **c** Latency of the different groups of mice in finding the platform in the MWM tests. n = 8 per group. **d** Average values of time the different groups of mice spent in the target quadrant and the other three quadrants (opposite, right and the left) during the probe test. n = 8 per group. **e** Distance travelled of the different groups of mice in finding the platform in the MWM tests. n = 8 each group. **f** Average values of swimming speed of the different groups of mice. n = 8 per group. **g** Left: representative traces of fEPSPs evoked in the CA1 by electrical stimulation of the Schaffer-commissural projection before and after HFS stimulation in the different groups of mice. Right: time course of fEPSP slope normalized to baseline. The fEPSP slope is plotted as a percentage change against the baseline (20 min) before HFS. HFS is indicated by arrows. n = 6 from 3 mice. **h** Normalized fEPSP slope (as a percentage of the baseline) in the hippocampus CA1 of the different groups of mice. n = 6 from 3 mice. Data are represented as mean ± SEM. *P < 0.05 based on unpaired t tests; ns, not significant (P > 0.05)

For investigating whether the neurological side effects of simvastatin are reversible, the medication was then weaned over a 4-week period in the simvastatin-treated mice. After that, the hippocampal cholesterol levels, LTP amplitude and the memory capacity were all re-examined in these mice. The hippocampal cholesterol concentration was restored to normal level testified by paDESI-MS imaging (Fig. 4a). Both the simvastatin-impaired recognition memory and spatial memory were significantly restored after simvastatin discontinuation (Fig. 4b–f). In addition, the LTP of fEPSP slopes in hippocampal CA1 slices were also recovered (Fig. 4g, h). These results suggest that the simvastatin-induced impairment of hippocampal cholesterol, synaptic plasticity and memory is transient and reversible.

## Discussion

Statins are widely known as a type of medications lowering low-density lipoprotein (LDL) cholesterol which is always referred to as bad cholesterol [28]. Emerging evidences suggest that statins may affect brain cognitive function [8, 29]. However, the underlying mechanism is still poorly understood. The data presented in this study provides several lines of evidence that BBB-permeable simvastatin may impair cognition via reducing hippocampal cholesterol. First, the long-term simvastatin treatment causes a significant reduction in hippocampal LTP, and leads to the inferior performance of MWM and NOR tests. Second, simvastatin reduces the hippocampal cholesterol concentration. The hippocampal cholesterol level is well correlated with the memory function of mice. Third, cholesterol discontinuation reverses the negative effects of simvastatin on hippocampal cholesterol level and synaptic plasticity. These results together suggest simvastatin may impair cognitive function by reducing cholesterol concentration in hippocampus. More importantly, the present study may provide some guiding significance for clinical practice. Although the effects of simvastatin are transient, patients requiring long-term usage of statins should select the BBB-impermeable drugs whenever possible, especially for patients with cognitive disorders.

In the present study, the paDESI-MS imaging technique is introduced to directly measure cholesterol concentration in hippocampus [26]. Generally, the cholesterol levels in biological tissues are determined usually by indirect measurements, such as classical chemical methods, enzymatic assay and analytical instrumental approaches including gas and liquid chromatography [30]. Compared with the conventional approaches, the paDESI-MS imaging exhibits several unique advantages. First, the paDESI-MS enable detecting cholesterol directly rather than indirectly measuring the H_2_O_2_ yielded from the oxidase-mediated oxidization of cholesterol [31]. Second, the MS imaging achieves the in-situ detection of cholesterol in specific subregions of the brain, allowing us to specifically measure cholesterol in hippocampus without interference from cholesterol-rich regions close to the hippocampus such as the corpus callosum. Thus, the paDESI-MS imaging is a powerful technique for qualitative and quantitative analysis of brain cholesterol.

Except for hippocampus, other brain regions may also be affected by simvastatin. Although our MS imaging tests only focus on the hippocampal brain area, cholesterol reduction in white matter and a few brain regions adjacent to hippocampus such as corpus callosum is also observed. Considering this, simvastatin may also affect other neurological functions such as motor and emotion. However, our present results indicate that simvastatin has no effects on motor ability and anxiety behaviors of mice. This is consistent with the clinical studies that no side effects on motor function and emotional states have been observed in patients treated with statins [32–37]. These negative results can be attributed to several reasons. For example, simvastatin may have a weaker cholesterol lowering effects in the brain regions related to motor function and emotional regulation when compared with the hippocampus. In addition, the compensation pathways for cholesterol synthesis in these brain regions may be activated after simvastatin administration. Thus, future studies should focus on the heterogeneity among different brain regions in cholesterol synthesis and metabolism. Notably, even in the hippocampus, various factors such as drug dose, duration of treatment and age may also differentially affect the effects of simvastatin. For example, previous studies have reported that hippocampal LTP could be enhanced when animals were administrated of a lower dose of simvastatin [38], when brain slices were treated with acute simvastatin incubation [39], or when older animals were used [40].

Statins including lipophilic statins and hydrophilic statins have different capacity to cross the BBB [6]. Cholesterol in the brain is locally synthesized independent from peripheral circulating cholesterol due to the presence of BBB [11, 41, 42]. Thus, BBB-permeable lipophilic statins may affect brain cholesterol synthesis and corresponding neurological functions. The present study shows that simvastatin reduces hippocampal cholesterol level and impairs hippocampal synaptic plasticity and memory function. Mounting evidence has reported that hippocampal cholesterol is correlated with learning and memory [19, 22, 43]. Increased cholesterol efflux impairs hippocampal synaptic plasticity and causes neurodegeneration [22]. Hippocampal cholesterol reduction impairs brain synaptic plasticity and leads to cognition impairment [23–25]. In addition, LTP formation has been evidenced to be mediated by many synaptic membrane proteins such as voltage-gated K^+^ channels, Na^+^ channels and Ca^2+^ channels, NMDA receptors and AMPA receptor [43–49]. Cholesterol has been widely reported to modulate the function of these ion channels [44, 48, 49]. Thus, simvastatin may affect the synaptic membrane fluidity and the function of ion channels in the synaptic membrane by lowering hippocampal cholesterol synthesis.

## Data Availability

All data in the current study are available from the corresponding author on reasonable request.
